# Proceeding of the Royal Society

**Published:** 1872-10

**Authors:** 


*Proceedings of the Royal Society.* Vol. XX. Nos. 130 to 134 inclusive.

The Transactions of this Society are as usual full of interest. In Number 131 we notice some interesting remarks by F. Le Gros Clark, Surgeon to St. Thomas, accompanied with a diagram, on the Mechanism of Respiration, and an address by A. Dupré on the Elimination of Alcohol. The balance of the papers are mostly of a purely scientific character, uninteresting to the general practitioner.

